# Management of Radix Paramolaris With a Distal Canal in the Mandibular First Molar: A Case Report

**DOI:** 10.7759/cureus.36167

**Published:** 2023-03-15

**Authors:** Priti Shukla, Varsha Sharma, Ravinder S Bedi, Shivesh Acharya

**Affiliations:** 1 Orthodontics, All India Institute of Medical Sciences, Raebareli, Raebareli, IND; 2 Pediatric and Preventive Dentistry, All India Institute of Medical Sciences, Raebareli, Raebareli, IND; 3 Dentistry, All India Institute of Medical Sciences, Raebareli, Raebareli, IND; 4 Pediatric Dentistry, All India Institute of Medical Sciences, Raebareli, Raebareli, IND

**Keywords:** root canal therapy, mandibular first molar, middle mesial canal, radix entomolaris, radix paramolaris

## Abstract

Mandibular first molars, also called six-year molars as they appear at around six years of age, are the first permanent teeth to erupt in the oral cavity. They are the teeth most commonly affected by dental caries. Anatomically, the tooth presents with two roots and three canals. In very rare cases, presence of an extra root or a supernumerary root has been associated with the tooth. When this root is present lingual to the distal root, it is called radix entomolaris whereas when it is present buccal to the mesial root, it is called radix paramolaris. The presence of veiled canals is possible due to variation in the anatomy of the tooth. It is therefore necessary to locate, prepare and obturate these hidden canals in order to achieve success in endodontic treatment.

## Introduction

Mandibular first molars are the first permanent teeth to erupt in the oral cavity; they are also the teeth most commonly affected by dental caries [1]. They usually present with two roots and three canals but variations have been reported. The presence of an extra root lingual to the distal root has been documented by Carabelli et al. as radix entomolaris; the one buccal to the mesial root has been reported by Bolk et al. as radix paramolaris [2-4]. Barker et al. reported the presence of a middle mesial canal as anatomic variation of the tooth [5]. The current case report is a less explored combination of radix paramolaris with the presence of two canals in the distal root.

## Case presentation

**Figure 1:**
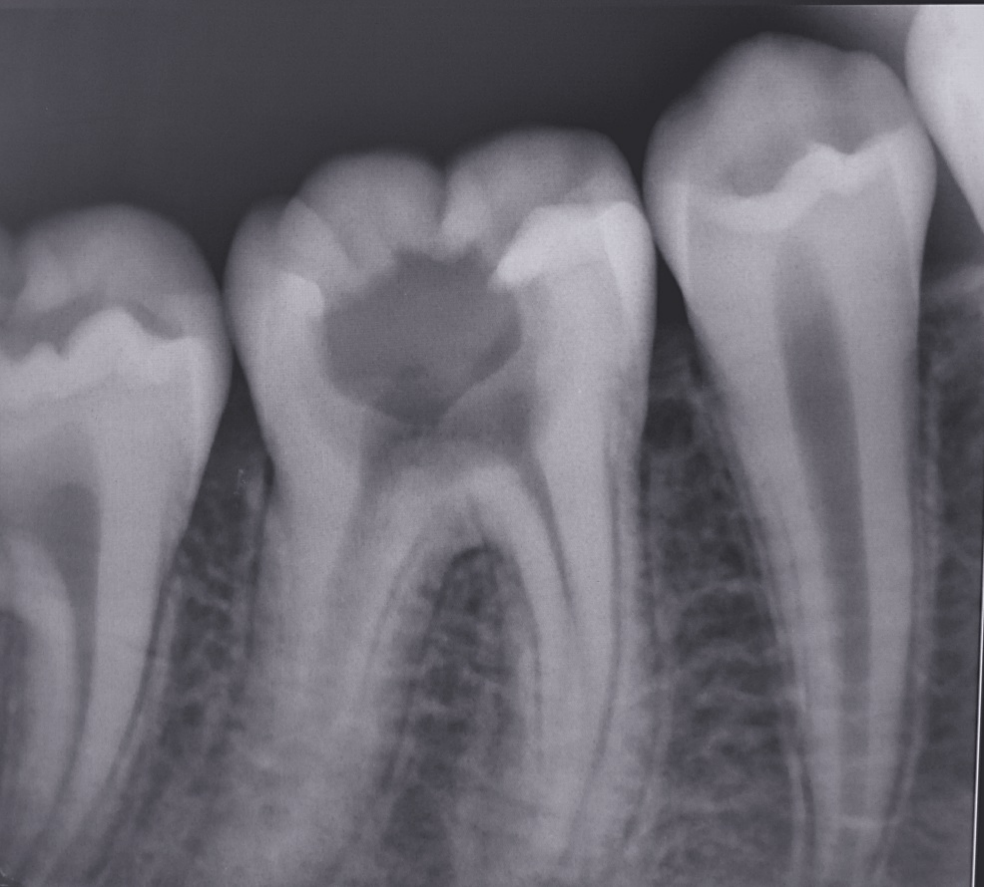
Pre-operative radiovisiography image

A 13-year-old male patient came to the Department of Dentistry with a chief complaint of dull pain in the right lower back region of his jaw since three days. Both family and medical histories were non-contributory. The clinical examination revealed deep dentinal caries in relation to the right mandibular first permanent molar. The affected tooth was tender on vertical percussion but asymptomatic on palpation. Upon thermal pulp testing, the patient experienced intermittent mild pain that remained for 15-20 minutes after the removal of stimulus. Hence, the cause of the pain was chronic irreversible pulpitis of the affected tooth. Radiovisiography (RVG) revealed radiolucency involving enamel, dentine and pulp without periapical changes (Figure 1).

An interesting feature seen was the presence of radix paramolaris or a third root in the mesial side of the tooth. The right inferior alveolar nerve was anaesthetized by 2% lignocaine and 1:200,000 adrenaline solution. After rubber dam placement, the carious portion was removed and access cavity preparation was done. Five canals were located: three in the mesial root and two in the distal. All the five canals had separate canal orifices and apical foramen. The pulp was extirpated.

**Figure 2:**
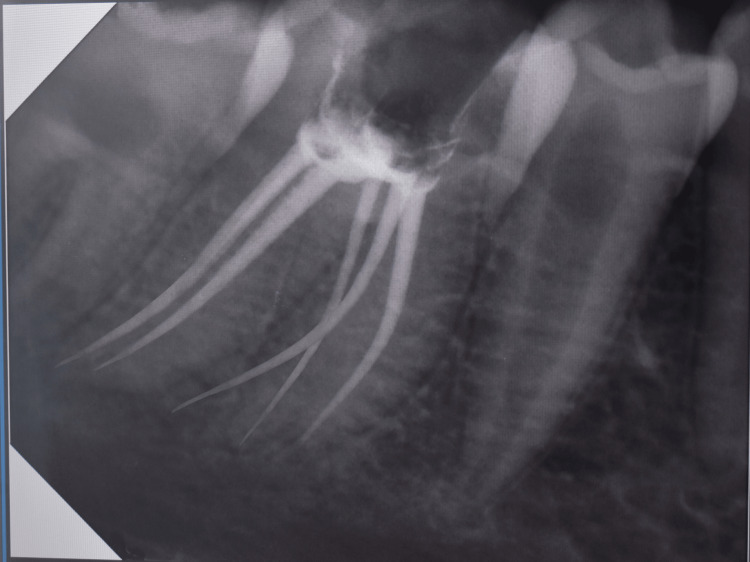
Post-obturation radiovisiography image

On the second sitting, the root canals were cleaned and shaped using hand-use protaper shaping files (Maillefer, Switzerland) till the F1 master file. Irrigation was done using 5.2% sodium hypochlorite. As the tooth was asymptomatic till a week later, the canals were dried with sterilized paper points and obturation was done using the protaper F1 gutta percha cone using lateral compaction technique. A radiograph was taken (Figure 2).

Glass ionomer cement restoration was placed to seal the access cavity and a porcelain-fused metal crown was placed. The patient was kept on a regular follow-up and was found asymptomatic on one, three and six months, post-operatively.

## Discussion

The mandibular first permanent molar can present with varied anatomy. The detection of canals is important for successful treatment and satisfactory long-term prognosis. Radix paramolaris can present as a separate mature root or as a conical extension. It has been further classified as type A in which the cervical part is located on the mesial root complex, and type B in which the cervical part is located centrally between the mesial and the distal root complexes [6]. The present case report is of type A radix paramolaris. Another anatomical variation observed in the documented case was the presence of two canals in the distal root, which is in agreement with the prevalence of 21.8% as reported by Bansal et al. [7]. As the RVG clearly showed the presence of three roots and as upon clinical instrumentation, following access cavity preparation, three different canals were located in the mesial segment and two canals on the distal segment, no cone beam computed tomography imaging was done.

## Conclusions

Anatomical variations in tooth structures are a little uncustomary yet present. When treating a case with root canal therapy, acknowledgement of such variations becomes important. It is thus essential to locate, prepare and obturate these veiled canals in order to achieve success in endodontic treatment. The knowledge of such variations like radix entomolaris and radix paramolaris will help clinicians to prevent procedural errors.
